# The changing forms and expectations of peer review

**DOI:** 10.1186/s41073-018-0051-5

**Published:** 2018-09-20

**Authors:** S. P. J. M. ( Serge) Horbach, W. ( Willem) Halffman

**Affiliations:** Faculty of Science, Institute for Science in Society, Radboud University Nijmegen, P.O. Box 9010, 6500 GL Nijmegen, The Netherlands

**Keywords:** Scientific integrity, Scientific misconduct, Peer review, Innovation, Scientific publishing

## Abstract

The quality and integrity of the scientific literature have recently become the subject of heated debate. Due to an apparent increase in cases of scientific fraud and irreproducible research, some have claimed science to be in a state of crisis. A key concern in this debate has been the extent to which science is capable of self-regulation. Among various mechanisms, the peer review system in particular is considered an essential gatekeeper of both quality and sometimes even integrity in science.

However, the allocation of responsibility for integrity to the peer review system is fairly recent and remains controversial. In addition, peer review currently comes in a wide variety of forms, developed in the expectation they can address specific problems and concerns in science publishing. At present, there is a clear need for a systematic analysis of peer review forms and the concerns underpinning them, especially considering a wave of experimentation fuelled by internet technologies and their promise to improve research integrity and reporting.

We describe the emergence of current peer review forms by reviewing the scientific literature on peer review and by adding recent developments based on information from editors and publishers. We analyse the rationale for developing new review forms and discuss how they have been implemented in the current system. Finally, we give a systematisation of the range of discussed peer review forms. We pay detailed attention to the emergence of the expectation that peer review can maintain ‘the integrity of science’s published record’, demonstrating that this leads to tensions in the academic debate about the responsibilities and abilities of the peer review system.

## Background

### Quality and integrity in science

Recently, there has been heated debate on the quality, credibility and integrity of scientific literature. Due to a perceived increase in scientific fraud and irreproducible research, some claim the publication system, or even science in general, to be in crisis [9, 25]. This rising concern has become obvious in the media, in policy initiatives, as well as in scientific literature. Concerned scientists as well as policymakers increasingly express their worry about data manipulation, plagiarism, or questionable research practices that affect the functioning of science [56].

A key issue in the debate on scientific integrity has been the extent to which processes of institutional self-regulation are able to track and prevent misconduct (e.g. [54, 108]). It has long been assumed that misconduct could hardly occur in the sciences due to well-established self-regulating mechanisms [64]. Sociologists of science in the tradition of Merton assumed that any form of research misconduct would sooner or later come to light due to scientists’ motivation to challenge competing knowledge claims via the peer review system, replication studies, or the presence of a whistle-blower, at least in as far as misconduct involves the misrepresentation of the research process [118].

The system of peer reviewing research papers in particular has long been central to these notions of self-regulation [57]. However, the expectation and ability of the peer review system to detect fraudulent and erroneous research is contentious and has developed and changed over time. While some currently argue that ‘safeguarding the scientific integrity of published articles’ is one of peer review’s core responsibilities [51, 67, 90, 108], others argue that the system was never designed, nor meant to do so [11, 105, 109]. Some even claim that peer review ‘ensures the state of good science’ and ‘assures that science is trustworthy, relevant and valuable’ [20, 113, 114], while others regard these claims as mere ‘myths’, and find peer review to be conservative, biased, and putting a burden on (unpaid and unrecognised) reviewers [11, 105, 108, 109].

Nevertheless, most scholars seem to agree that peer review serves as a filter in distinguishing between ‘good’ and ‘bad’ science [86, 110]. Despite an ever-growing number of concerns about its effectiveness, fairness and reliability [28, 38, 71, 72, 105, 109, 114, 116], peer review is still considered the best available practice to ensure the quality and correctness of the scientific literature. However, the devil is in the detail: specific features have been added to the peer review process in the expectation they would address specific problems obscured by blanket notions such as ‘quality’. Currently, there is a clear need for a systematic analysis of peer review forms and their underlying concerns, especially in light of a wave of experimentation fuelled by new internet technologies.

Ever since being established, journal peer review has developed in a quite disorderly fashion so that currently it comes in many shapes and sizes [16, 110]. For various reasons, different journals and publishers tend to adhere to different forms of peer review. Among others, the increased specialisation in areas of science [11, 90], the rapid growth of science [15, 112], the changing financial foundation and incentives in scientific publishing [49, 51, 67, 69] and the advent of novel technological possibilities [12, 50, 69, 106] all have had a major impact on the structure of peer review. By now, so many forms of peer review exist that some claim we can no longer call it a single system [11, 86, 90]. While peer review is used in many contexts, including in grant assessment and career advancement, we will focus here on peer review of journal articles only. In addition, we will focus on the aspects directly affecting the review of a paper’s content in the editorial process (i.e. the intellectual exercise), rather than on the (technical) infrastructure that facilitates it (i.e. contemporary digital review submission systems or the analogue predecessors in which reviews were communicated via e-mail.)

**Table 1:** Forms of peer review blinding

	Reviewer		
Author		Anonymised	Identified
	Anonymised	Double-blind	‘Blind review’
	Identified	Single-blind	Open review

This article has three objectives. First, we describe the diversity of current peer review practices and innovations in the section ‘Main text—the historic development of peer review’. We review the academic literature to analyse the various rationales for developing these new forms, and discuss how they have been implemented. In doing so, we add some of the latest innovations to a new overview that improves on existing ones. Second, using our updated overview, we will identify some common patterns in the various peer review forms in a typology that systematises this diversity. This typology, presented in the section ‘Diversity of forms’, can serve as a useful tool for future research on peer review instruments, e.g. in considering the quality and effectiveness of review forms. Third, in the section ‘Main text—diversity of expectations’, we will pay detailed attention to the emergence of novel expectations some have of peer review, specifically for maintaining ‘the integrity of science’s published record’. We will also indicate how these expectations have inspired peer review innovations.

We will demonstrate that these new expectations are not always entirely compatible with one another and hence lead to tensions in the current academic debate about what peer review can and should do. Underlying this debate, we note a growing expectation that the scientific literature will serve as a database of established knowledge, rather than as a collection of research reports, pointing to more fundamental disagreement about the nature of scientific knowledge. At least some of the expectations of peer review are not just about the practicalities of ‘how to make it work better’; many also expect the process to address the functions of the publication system and even what it means to publish an account of a research project.

## Main text—the historic development of peer review

### The appearance of peers

Many accounts of the peer review process’ origins locate its beginnings in the seventeenth century, coinciding with Henry van Oldenburg’s establishment of an academic journal [11, 16, 66, 90]. However, historians of science have increasingly rejected this claim. In fact, they argue that many journals did not introduce peer review in the sense of ‘peers judging the publishability of a manuscript’ until after the Second World War [6, 7, 42]. Earlier, decisions on acceptance or rejection would commonly be made by a single editor or a small editorial committee, frequently based on their personal preferences [6]. In fact, the term ‘peer review’ only emerged in the scientific press in the 1960s and even then was initially used to describe grant review processes, rather than journal article reviewing [7, 77].

The practice of assessing or commenting on manuscripts prior to publication primarily arose in learned societies in the early and mid-nineteenth century [77]. In their early forms, reviews were commonly performed by other society members and hardly intended to act as a gatekeeping mechanism. Instead, comments or reports about manuscripts were aimed, for instance, at increasing the public visibility of science or evaluating new findings in service of the king [21]. Only in the late nineteenth century, by the time some review practices were well-established [77], was the referee gradually ‘reimagined as a sort of universal gatekeeper with a duty to science’ [21]. Despite some early concerns, the system remained in use and was slowly adopted by independent journals, also outside the scope of academic societies.

In the late nineteenth century, the *British Medical Journal* (BMJ) was one of the independent journals to pioneer the novel practice of using external reviewers to assess submitted manuscripts. Since 1893, its editor-in-chief, Ernest Hart, called upon the specialised knowledge of a reviewer, whom he labelled as ‘an expert having knowledge and being a recognised authority in the matter’. Although Hart acknowledged the fact that such a system was labour intensive, requiring ‘heavy daily correspondence and constant vigilance to guard against personal eccentricity or prejudice’, he believed that his system of selecting outside reviewers was ‘the only system that seems adequate to the real needs of professional readers’ [16].

In bringing outside expertise to the review process, extending its scope to actual peers, rather than a closed group of editorial committee members, the peer review process began to take the shape that is still very common today. However, this system of employing other peers than the journal’s or publisher’s committee members only became regular practice after the Second World War [7], with a major journal such as *Nature* adopting such a peer review system as late as 1973 [6].

In addition, differences between scientific fields were substantial. From the outset, (external) reviewing practices were considered time-consuming, costly and labour intensive. Especially in fast-developing fields, peer reviews were considered so burdensome that they prohibited quick knowledge exchange, and so made journals reluctant to use review mechanisms akin to those in learned societies [5, 77]. Moreover, different publishing formats, e.g. monographs as opposed to journal articles, have resulted, even today, in distinct review practices in different research fields [77, 86].

Several factors have been at the heart of journals’ and societies’ rationales for starting to use external reviewers in their review practices. Specialisation and growth in science were two such motivating factors. As growing numbers of manuscripts covering a wider range of topics and specialisations were submitted, editors had to select which they would publish and were less and less capable of judging all submitted work themselves. This led to them soliciting external, expert opinions [11, 16, 74]. Other factors, including a shift in the role of science in society, could have been equally important in establishing review systems. Specifically, the practice of external referees assessing and judging submitted manuscripts was taken up most prominently in the UK and North America, while other regions remained very hesitant until well after the Second World War [21]. And then, even between the UK and USA, there are differences. In the USA, review practices were perceived (among others) as mechanisms for providing scientific legitimacy that would answer to growing requirements of public accountability. These expectations were less pronounced in other regions, which partly explains the slower development of external review systems [6, 21]. However, the gradual spread of publications being peer-reviewed as a quality indicator supervised by research managers provided a strong incentive for researchers to publish in peer-reviewed journals.

In spite of currently being revered in some sciences, peer review still has a remarkably short history. The work of luminaries such as Einstein, for example, was often published without being peer reviewed [61]. Peer review practices were varied and often contentious. In the debates on peer review, specific concerns led to innovations and modifications, to which we will now turn our attention.

### The concern for fairness and bias

#### Blind justice

After the system using external reviewers became widely implemented in the 1960s and 1970s, developments in peer review succeeded each other with increasing speed. The first major developments concerned the level of anonymity in review. Initial peer review practices (nearly) always disclosed authors’ identities to editors and reviewers, whereas authors knew the identity of the editor-in-chief, but not necessarily of the editorial committee or invited outside reviewers [77]. Already in the 1950s, in the framework of sociology journals, the matter of blinding authors’ and reviewers’ identities was raised. The *American Sociological Review* was the first to install regulations in which authors were required to attach a detachable cover page to their manuscript so that their identities could be obscured. The rest of the paper had to ‘bear the title as a means of identification, but not name and institution’ [2]. From sociology, the anonymization of authors spread to other social sciences and the humanities.

Starting in the 1970s and continuing to the present, various researchers have examined the bias in selecting and accepting manuscripts of authors of different demographics and status [119]. In response to this debate, various categories describing different forms of author and reviewer anonymity in peer review were established in the mid-1980s [85, 88]. These categories are still in place and frequently show up in discussion regarding peer review (Table 1):

The single-blind and double-blind systems have continued to be the most common forms of evaluating articles, with a tendency to use the single-blind format in the biomedical and natural sciences, and a the double-blind system more frequently in the social sciences and humanities [85, 113, 114]. In addition, a *triple-blind* review process has been proposed, in which the identity of the author is not only concealed from the reviewers, but also from the handling editors [94]. Currently, a few journals use this system, but it remains fairly uncommon in designing review processes [110].

The rationale for developing the system of double-blind review was simple: in the new system, only the journal’s secretariat would know the author’s identity; therefore, peer evaluation and editorial committee decisions would rely only on the content of the manuscript and not on the reputation of the author or his/her institute [85]. Subsequently, when author anonymisation spread to other social sciences and humanities, a different rationale emerged. The extension was introduced not only on editorial initiative as had been the case when the *American Sociological Review* established the system in sociology, but also resulted from demands for fair and equal treatment of minority groups in science, most notably women [10]. As such, this development is part of a broader societal movement, including the second feminist wave, which demands equity between different members of society [115].

The call for more equal treatment of minority groups was strengthened by various assessments of bias in peer review. Although evidence of such bias remains slightly indecisive [110], there are strong indications that it exists, especially regarding gender and status/affiliations. This was confirmed in a famous study by Peters and Ceci [83], in which they resubmitted published manuscripts with different authors’ and institutions’ names and paraphrased titles to the very same journals that had published them. The vast majority of the manuscripts (8 out of 12) was rejected on grounds of poor quality or ‘methodological flaws’ [83]. Similar effects were reported in later studies [80, 96]. The initial report by Peters and Ceci initiated a fierce debate, with dozens of letters in response. Specifically, the perception that manuscripts were judged not merely on their content, but also according to ‘circumstantial’ factors such as the author’s affiliation, background and personal characteristics invoked debate leading to the spread of double-blind review [85]. This format of review now presents a way of combatting referees’ bias. However, in the digital age, critics have repeatedly pointed to the ineffectiveness of blinding author identities as a simple Google-search commonly enables identifying the authors of a ‘blinded’ manuscript.

#### Transparency: in reviewers we trust?

Interestingly, the issue of reviewer bias as a threat to the quality and fairness of peer review has not only led to the establishment of double-blind peer review, but also to its radical opposite: the system of open review. Currently, the term ‘open review’ is used for many different models and encompasses a wide variety of characteristics of peer review. A recent systematic review of the definitions for ‘open peer review’ demonstrates that scholars use the term to indicate processes in which, among others, the identity of the authors and reviewers are public, the review reports themselves are openly available, or the review process allows reviewers and/or authors to interact with each other [95]. In this paper, we use the term ‘open review’ merely to indicate that the identity of the authors and reviewers are mutually known to each other.

Open review gained momentum in the late 1990s, with the decision of the *British Medical Journal* to publish both reviewer names and reviews [104]. Other initiatives followed, most notably in the biomedical sciences [3].

The rationale for choosing an open system of peer review is transparency. Its advocates argue that open review leads to more constructive feedback, reduces reviewers’ bias and gives credit to the reviewer [46]. Thereby, it addresses some of the same concerns as those raised by the double-blind format, but with a radically opposite strategy. In addition, open review could reduce the chance of reviewers taking unfair advantage of their position as reviewer, either by plagiarising the manuscript under review, unjustly delaying its publication or advising rejection for unjust reasons [46, 86, 110, 112].

The system of open peer review claims to contribute to reviewer evaluation, in response also to questions regarding the integrity or fairness of reviewers, rather than the integrity or quality of the evaluated manuscript. This is especially pertinent in systems that communicate reviewers’ identities not only to the authors, but also to the general readership. In addition, formats of open review, in which the review reports are published alongside the article, provide another measure to increase transparency and therefore invoke scrutiny of reviewers. The emergence of the open review format hence allows surveillance of a system that has criticism as its major task.

In contrast, opponents of the system have stressed that open review could pose a threat to the quality of reviewing. This would especially be a concern when junior researchers are to review manuscripts by more senior colleagues, fearing professional reprisal if they submit negative reviews. In general, scholars have expressed concern about reviewers being milder in open review forms, thereby leading to more and, potentially poorer, manuscripts being published [95].

### Technological advances in peer review

From the 1990s onwards, various technological advances paved the way for novel development of the peer review system. This opened possibilities which include new timing of the process, such as post-publication peer review (see 2.3.1); publishing more articles, while allowing a shift of review criteria from importance to rigour (see 2.3.2); the advent of automated checks and similar software tools (see 2.3.3); further specialisation of peer review (see 2.3.4); and more communication during the review process (see 2.3.5). Using these headings, we will attempt to describe the bewildering experimentation that erupted in the age of the internet. As we will show, these changes were not just driven by technological possibilities, but also by the interplay between technological potential and specific concerns about peer review’s imperfections.

Even so, besides opening up possibilities for a wide range of novel peer review formats, arguably, the most important development brought on by the advent of digitization, lies in the technical infrastructure facilitating review. This mainly affected the possibility of contacting and finding suitable reviewers much more quickly than before. Accessing researcher’s webpages and email addresses allowed for much faster circulation of manuscripts and review reports, potentially increasing the speed and efficiency of the review process enormously. In the remainder of this section we will focus on the intellectual aspects that, facilitated by new technologies, affect the actual review process.

#### The timing of peer review in the publication process

Traditionally, peer review occurs between the submission and publication of a manuscript. In this format, editors receive a manuscript and possibly send it to outside reviewers or an editorial committee, who advise whether a manuscript is good enough to be published. Over the last two decades, two new forms of peer review have emerged that change the chronology of the reviewing. Firstly, there is a format in which manuscripts are evaluated after publication, the *post-publication peer review*, and secondly, a system in which articles are reviewed prior to submission to the journal, a format called *registered reports*.

##### Post-publication review and preprint servers

In the 1990s, several studies demonstrated that peer review is potentially biased, slow, unreliable and inconsistent (e.g. [28, 29, 72, 83]), thereby nourishing the desire for alternative models and the formation of preprint archives. Especially, the system’s indolence and inconsistency were indicated as reasons for the formation of post-publication peer review. Preprint servers were established, based on already existing archives of print-based mail exchanges in high-energy physics. Even though some forms of disseminating preprint articles have been in place since the 1960s [70], the advent of the internet and digital technologies enabled the establishment of large and fast-operating archives in which authors could freely upload their manuscripts, thereby bypassing publishers. In these archives, manuscripts usually go through a minor evaluation to check whether they meet minimal standards of academic writing [50, 112]. Subsequently, the actual review is done by community members who comment on the manuscript, either via personal or public communication. Authors can then improve the manuscript and upload new versions to the archive [14, 50]. Originating in physics, astronomy and mathematics, the preprint servers have found their way to other scientific disciplines, with similar servers set up for biology, engineering and psychology [110].

At first, these preprint servers were mainly used by authors to make preliminary versions of their articles available, before submitting the final version to a peer-reviewed journal. However, with the enormous increase in submissions to preprint archives recently [112], these servers have themselves become a major communication channel in, which some authors use as a sole venue for their manuscripts [36]. This fast dissemination method allows scholars to keep up with each other’s work, provides a way of crediting the first author(s) for presenting novel findings and thereby solving priority issues, and allows readers to comment on early drafts of a paper. Ideally, this results in exchanging ideas and improving the manuscript [14, 50]. However, despite an increased number of papers being deposited in arXiv and other preprint servers, the proportion of scientific literature made available in this fashion is still very low and limited to only a few academic fields [112].

Besides being used in preprint servers, post-publication review has gradually also been taken up by journals and publishers. The first journal to implement this format was *Electronic Transactions in Artificial Intelligence* in 1997 [36, 87]. Introducing this new review form served mainly to accelerate knowledge distribution. Especially in the last few years, a number of journals have switched to this post-publication model of peer review. Finally, several independent platforms such as *PubPeer* were established, in which post-publication review of any published manuscript can be done, independent of what kind of review it went through during the publication process [62]. These platforms will be discussed in more depth in the section ‘Novel actors and cooperation in the review process’.

Besides responding to concerns of speed and consistency, introducing open archives resulted in several new expectations of peer review. Rather than being a selection or gatekeeping mechanism, according to some scholars, reviewing should be transformed into a filtering process that presents relevant literature to researchers in the right fields: ‘… peer review needs to be put not in the service of gatekeeping, or determining what should be published for any scholar to see, but of filtering, or determining what of the vast amount of material that has been published is of interest or value to a particular scholar’ [37]. Hence, the peer review system should not be thought of as a way of stopping ‘irrelevant’ research from being published, but merely as a way of directing the right literature to the right reader. By lowering the threshold for publishing manuscripts, including those reporting negative results, this system also serves as a response to the apparent bias in published manuscripts towards positive results [27]. Some consider countering this bias an important measure to restore the integrity of the scientific literature [111].

The system of publishing articles prior to being reviewed serves to enhance research integrity in two additional ways. Firstly, the publication of preprints can improve the detection of fraudulent research. There are several cases in which authors, often after previous rejections from journals, alter their data and/or conclusions to deliver a more positive result. Such cases of spin or data manipulation are more easily detected if preprints of a manuscript have been published. In this way, preprints serve as a means of detecting authors’ improper behaviour. Secondly, preprints also serve a function in recognising reviewer misbehaviour, such as plagiarising manuscripts under review or delaying review to obtain an advantage in priority issues.

Besides these advantages, establishing preprint servers and introducing electronic publishing in general have had a major effect on the costs of publishing and of obtaining access to scientific literature. Continuing a trend started by large publishing companies that created a publishing market in the 1980s, the introduction of electronic publishing in the mid-1990s brought a massive increase in the number of journals, articles and citations [69]. This number shows a concentration of articles and citations in the outlets of large commercial publishers. In the fields of both medicine and natural science, as well as in the social sciences, large commercial publishers bought journals from smaller publishers and established new journals themselves, in order to drastically increase their market share in academic publishing [41]. One of its consequences has been a sharp increase in journal prices and the establishment of ‘big deals’ with (university) libraries [69].

##### Registered reports

A second major development regarding the timing of peer review in the publication process has been the establishment of the registered reports system, first introduced by the journal *Cortex* in 2013 [17, 76]. In this form of peer review, which is still restricted mainly to medical fields and psychology, manuscripts are usually reviewed in two stages. The initial and most important review stage takes place after the study has been designed, but prior to data collection. At this stage, only the rationale for undertaking the research, the research questions and the research methodology are reviewed. On the basis of these criteria, a study is either accepted or rejected, before any data has been collected. In the subsequent stage, after data collection and analysis have taken place, authors compose their manuscript by adding their results and conclusions to the registered report. The final manuscript can then be reviewed on the basis of consistency and adequately having drawn conclusions from the data. Taking this further, *BioMed Central (BMC) Psychology* recently published the first articles that had been through a completely ‘results-free review’, in which the second phase of peer review was entirely omitted [19].

The main reason for introducing registered reports lies in the alleged ‘replication crisis’ in several areas of science. Registered reports are a means of making the execution of replication studies more attractive: ‘Peer review prior to data collection lowered the barrier to conduct replications because authors received editorial feedback about publication likelihood before much of the work was done’ [79]. Generally, many journals are reluctant to publish replication studies, which potentially deters scientists from performing them: ‘If journals will not publish replications, why would researchers bother doing them?’ [79]. Prior clarity about publication chances based on research design, and not on the novelty of results, could encourage replication studies. In addition, registered reports can alter incentives for authors and reviewers to act with more integrity, in the sense that methodological accuracy and transparency become more important than pleasing possible readers: ‘Because the study is accepted in advance, the incentives for authors change from producing the most beautiful story to the most accurate one’ [18] and ‘review prior to data collection focused researchers and reviewers to evaluate the methodological quality of the research, rather than the results’ [79]. Hence, contrary to innovations that are mainly designed to allow additional scrutiny of the reviewer, registered reports address the integrity of the author and promise to reduce researchers’ rewards for dubious behaviour.

#### The changing peer review criteria

Besides yielding the system of pre-print archives, the advent of the internet and large databases further enabled journals to publish nearly unlimited numbers of articles. Novel publishing strategies and related peer review models became possible. A major development in this respect came with the launch of the open access journal *PLoS ONE*, by the *Public Library of Science* (PLoS), in 2006. In this journal’s review process and business model, reviewers are asked to base their recommendation for acceptance or rejection purely on the soundness and validity of the research, comprising the methodology, soundness of results and reporting. According to the journals’ philosophy, reviewers should not judge the novelty, relevance, or importance of research, which should be left to the reader and wider community [52]. By focussing on rigour and (ethical) soundness of research, the journal aims to ensure that useful results will all be published, and to prevent subjective assessment of a study’s importance or relevance.

Since its launch, *PLoS ONE* has been one of the most rapidly growing publication venues. In 2013, it published over 30,000 articles [24, 48], turning itself into the largest open access publisher and one of the largest scientific journals worldwide. Subsequently, other journals and publishers, such as *BMJ Open* and *SAGE Open*, have adopted the same non-restrictive review model [52].

These changes in review criteria content and in how they select have their roots in discussions on scientific integrity. Several motives have prompted *PLOS* and other outlets to focus on rigour and soundness of research [13, 84, 98]. First, it ensures the publication of all ‘valid’ research, irrespective of the study’s perceived importance by reviewers. This, among other things, facilitates the publication of replication studies and negative results [13]. In addition, the journals aim to deter authors from overstating results or otherwise engaging in questionable research practices in order to meet reviewer standards of importance. This review format was therefore partly set up to promote scientific integrity, not so much by increasing the detectability of fraudulent research or misconduct, as by stimulating scientific integrity from the outset [52]. However, this system could unintentionally also create new concerns regarding the literature’s integrity, for instance by overloading it with research of little relevance, or by creating incentives and opportunities to publish (irresponsibly) high numbers of articles.

Partly due to the less restrictive review process, the number of papers published in outlets employing this non-restrictive review model has grown rapidly. As a result, new challenges have emerged in the publication process. One of them is finding enough qualified reviewers to handle all submissions. For example, by 2014, *PLoS ONE* used more than 70,000 reviewers to process all submissions and the average review time drastically increased since *PLoS’*s launch in 2006 [24, 48]. In addition, the high number of published articles generates a growing concern about the scientific literature becoming unmanageably large, resulting from an abundance of articles many of which add little to the stock of knowledge. At the least, this creates a growing need for further filtering to ensure researchers can cope with the enormous number of potentially interesting papers. Novel systems will need to be established to draw readers’ attention to articles that are most likely to be useful to them.

#### Introduction of software tools to the review process

In addition to the possibilities of preprints and virtually unlimited numbers of publications, the advances of the internet and new digital technologies also offered dedicated technical support to assess whether papers are publishable. Technical assistance in various formats has by now become standard practice and most certainly will be extended in the (near) future [12]. The first major technical assistance to be implemented in peer review was plagiarism detection software. Copying text from various sources became easier than before once electronic publishing was introduced, and with internet assistance added concerns about plagiarism spread throughout academia, regarding student papers as well as research articles [4]. However, the first versions of plagiarism detection tools originated in the context not of textual plagiarism, but the copying of parts of programming code [35]. Only in later phases did this evolve into plagiarism detection tools for journals to recognise unwarranted copying in research articles [33]. Currently, the vast majority of journals and publishers use some form of plagiarism detection tool to assist in peer review [30], the *CrossCheck* system being the most common [117].

Besides assisting with plagiarism detection, online tools have recently come to assist reviewers in several other ways. Most notably, some automatic analysis that checks for the correct use of statistics in manuscripts has been introduced [32]. Aided by artificial intelligence technologies, software protocols have been developed to assess completeness, consistency and validity of statistical tests in academic writing, thereby specifically targeting the (intentional) misuse of statistics in research, which some believe to be a major factor in the alleged integrity and reproducibility crisis [78]. Additionally, the assistance of software in detecting image manipulation, which is considered an increasing form of fraud in various research areas, has successfully been implemented by several journals [100]. However, we should note that the use of image and statistics scanners is still rare and limited to specific research areas, most notably the medical sciences, physics and psychology.

In the future, automated computer software could well play an even more substantive role in the review process. Aided by machine-learning techniques, it has already become possible to check for bad reporting (failing to report key information or inconsistencies in reporting), data fabrication and image manipulation. In addition, Chedwich deVoss, the director of *StatReviewer,* even claims: ‘In the not-too-distant future, these budding technologies will blossom into extremely powerful tools that will make many of the things we struggle with today seem trivial. In the future, software will be able to complete subject-oriented review of manuscripts. […] this would enable a fully automated publishing process – including the decision to publish.’ [12] Although one should have some reservations on such predictions of a technological future, they do reveal some of the current expectations for peer review.

The implementation of software-aided detection mechanisms requires us to increasingly distinguish the ‘peer review process’ from ‘peer review’. Due to digital technologies and software tools normally not being imposed on the reviewer, but handled by the journal’s staff or editorial team, the review process now entails much more than individual reviewers merely doing quality assessment. Therefore, the use of these tools should be considered an additional step in the review process, rather than an integral part of the actual review by a ‘peer’.

In sum, digital technologies and software tools based on machine learning and artificial intelligence have been incorporated in some parts of the peer review process. Their primary use currently is to detect plagiarism, text recycling and duplicate publication; to analyse and review statistics and statistical analysis in specific fields; and to a lesser extent to detect figure or data manipulation [12, 32, 41, 110]. All of these clearly target the integrity of research and authors under review and specifically target those practices that have traditionally been labelled as outright fraud, namely falsification, fabrication and plagiarism. Hence, these digital technologies are a primary example of innovations in peer review specifically targeted to increase the detectability of fraudulent or erroneous research.

#### Novel actors and cooperation in the review process

Over the past decades, new actors have joined the review process, thereby compelling peer review itself to become more specialised. This applies to its content, for example introducing specialised statistical reviewers, as well as to the process, with commercial parties specialising in the reviewing process.

##### Statistical review

During the second half of the twentieth century, the use of statistics in research articles has drastically increased, especially in medical and psychological research [1]. The use of ever more complex, statistical models raised concerns about the validity of some statistical methods. In response to the publication of reviews demonstrating that published articles often report statistically unsound analyses, journals and publishers set out to dedicate more attention to statistical analyses in their review processes. From the 1960s onwards, several journals included specialist statistical reviewers to judge the soundness and quality of methodology and statistics in submitted manuscripts, again mainly in medicine and psychology [1, 101].

Despite repeated demonstration of widespread statistical and methodological errors in (medical) research, increasing the use of specialist reviewers to check for such errors has been slow. A 1985 survey of journals and publishers showed that only a very small proportion of journals paid specific attention to those factors in their review process [45]. Fuelled by current issues regarding research reproducibility and replicability [58, 78], many still agitate for intensifying the scrutiny of statistics. One consequence was the formation of a project called SMARTA, which brings together members of international statistical societies to assess the use of statistics in biomedical literature [47]. Such developments may well lead to statistics being given more attention in review, and even to further specialisation of reviewers.

##### Commercial review platforms

Besides the introduction of specialist statisticians to the review process, a new set of refereeing bodies has recently emerged [110]. In these new initiatives, review is dissociated from the journal in which the article is published. Several formats have emerged, of which one arranges the reviewing of articles prior to publication by independent third parties. Platforms such as *Peerage of Science*, *RUBRIQ* and *Axios Review* [82, 92] provide tools and services to conduct reviews and forward submitted manuscripts along with referee reports to a journal. In this way, reviews can be done faster and more efficiently, also by reducing the likelihood of a manuscript going through multiple reviews for various journals.

Notably, one of the commercial services providing independent review, *Research Square*, specifically focuses on the promotion of scientific integrity with the assistance of software tools. The platform attaches badges to manuscripts that pass various tests addressing specific ‘aspects of a research manuscript that [are] critical for ensuring the integrity and utility of the scholarly record’ [91]. It awards such badges after an ‘integrity precheck’, ‘statistical check’, ‘figcheck’ and ‘sound science check’, to name just a few. Thereby, the platform explicitly claims that such assessments can indeed be made as part of the peer review process. In a pilot study on submissions to two medical journals, *Research Square* actually reports detecting integrity issues much more frequently than would be expected considering current estimates on the extent of misconduct in science [81].

In addition to the systems providing pre-publication review, other independent platforms have emerged, such as *PubPeer* [89], in which any reader can comment on any published manuscript. These systems constitute examples of post-publication review independent of journals and publishers. These new trends have increasingly widened the definition of a *peer*, so that the term now refers not only to a small cluster of editor-selected experts, but to anyone who feels capable of understanding and evaluating a given piece of research. This emergence of an ‘extended peer community’ gives rise to novel challenges concerning the role of expertise in peer review, as well as to questions regarding who has the right and competence to judge the quality, soundness and relevance of scientific research [40]. In addition, some scholars have expressed concern about the role of public forums in signalling cases of problematic research, as this can lead to stigmatising researchers without them having due opportunity to defend themselves.

##### Cooperation in review

Another way of reducing the burden on peer review lies in the concept of ‘cascading peer review’. This model, which was first consistently used at the beginning of the twenty-first century, became common practice in the *BMJ* journals in 2010 [23] and is now widely used, especially by larger publishing houses. The system aims to avoid final rejection of a manuscript after peer review by redirecting critically reviewed manuscripts to potentially more suitable journals. In practice, larger publishing houses often use this system of redirecting manuscripts that are rejected for publication in top-tier journals to lower-tier journals within their portfolio. However, currently, peer review consortiums are formed to facilitate the practice of cascading review in smaller publishing houses as well [8]. The system of cascading reviews responds to the growing expectation of the review system to not necessarily act as a gatekeeper, but rather serve as a mechanism to direct relevant research to the right audience. As the system of cascading reviews is designed to avoid final rejection, it potentially focuses on the relevance of a manuscript, rather than its soundness, quality or integrity. This could have major implications for the scientific publishing system. Low rejection rates can raise questions about the veracity of knowledge, tolerance for ‘alternative facts’ [103] and rating the value of publications in research career assessment.

Both of these peer review models, cascading review and review by third parties, are designed to assure that one single manuscript does not have to go through multiple rounds of peer review. Sharing review reports, either from a commercial party or from a rejecting journal, with a potentially interested journal, decreases the number of reviewers assessing a single manuscript [8, 110]. This answers to a concern of the past few decades, that the peer review system is getting overloaded [65]. In addition, automatically (re-) directing manuscripts to the most suitable journal after review could reduce perverse incentives for authors, such as rewarding work in which conclusions are overstated to get the study published. On the other hand, it could also work in the opposite direction in that relaxing review standards might tempt authors to neglect nuances in the confidence that their work will eventually get published somewhere anyway.

#### New openness: discussion during review

Finally, the advent of digital technologies has paved the way for new levels of openness in the review process. Some journals, most notably journals at EMBO (*European Molecular Biology Organization*) and the *elife* journal, have attempted to improve editorial decision making by introducing interactive stages in the review process, during which reviewers and editors can share or discuss their reports and opinions on a manuscript before communicating a final decision to the author [31, 99]. In 2011, the *elife* journal pioneered this new model, referring to movements concerning transparency and accountability in peer review as rationale [99]. Later, other journals followed suit, partly related to the open science movements in which review reports are not only shared among reviewers, but also with the general readership.

The *Frontiers* journals launched in 2013 later established a more radical variant of this peer review model, labelled the ‘collaborative peer review’. This process set up a review forum for interaction between authors and reviewers. Such forums serve as an interactive stage in the review process, during which authors and reviewers discuss the paper online until they reach agreement on the most effective way to improve its quality [39, 52].

### Diversity of forms

Concluding from the overview in the previous subsections, the diversity of peer review forms has clearly increased significantly over the past few decades, thereby also diversifying the practice of quality control in research.

Structuring the discussion in the preceding subsections, the distinguishing attributes of various review forms can be classified along four dimensions, namely the selection conditions, the identity and access among actors involved, the level of specialisation in the review process, and the extent to which technological tools have been introduced. Each of the attributes has a range of possibilities, as presented in Table 2. The typology discloses a clear ordering of the current variety in peer review, providing a solid foundation for further research on, e.g., how often various forms are used, or how various peer review forms relate to other properties of the publication system.

**Table 2:** Forms of peer review categorised by dimension and attributes

Dimension	Attribute	Range
Selection conditions	Timing	a. No review
		b. Pre-submission (including registered reports)
		c. Pre-publication
		d. Post-publication
		e. Mixed methods
	Selectiveness	a. Merely access review
		b. Non-selective review
		c. Selective review
Identities and access	Type of reviewer	a. Editor-in-chief
		b. Editorial committee
		c. External reviewers selected by authors
		d. External reviewers selected by editor(s)
		e. Wider community / readers
		f. Commercial review platforms
	Anonymity of authors	a. Author identities are blinded to editor and reviewer
		b. Author identities are blinded to reviewer but known to editor
		c. Author identities are known to editor and reviewer
	Anonymity of reviewers	a. Anonymous reviewers
		b. Reviewers identities are open to the authors
		c. Reviewers identities are open to the reader
	Availability of review reports	a. Review reports are openly available
		b. Review reports are only available to authors, editors and reviewers
	Interaction	a. No interaction between authors/reviewers
		b. Reviewers only: interaction among reviewers
		c. Authors and reviewers: interaction between authors and reviewers
Specialisation in review	Structure	a. Unstructured
		b. Semi-structured: Reviewers are guided by some open questions or are presented with several criteria for judgement
		c. Structured: Review occurs through mandatory forms or checklists to be filled in by reviewers
	Statistical review	a. Not applicable
		b. Incorporated in review
		c. Performed by additional, specialist reviewer
		d. Performed through automatic computer software
	Training	a. No specific guidance or training available to/required from reviewers
		b. Guide for reviewers available (either online or on paper)
		c. Training/courses available for/required from reviewers
		d. No specific guidance or training available to/required from reviewers
Technological tools in review	Technical support	a. Review is performed manually
		b. Technical assistance in the form of plagiarism check
		c. Technical assistance in statistical review
		d. Technical assistance in image manipulation
		e. Other Technical support (e.g. machine learning techniques to assess consistency and completeness)
	Reader commentary	a. No reader commentary facilitated
		b. In channel reader commentary facilitated
		c. Out of channel reader commentary facilitated

## Main text—diversity of expectations

### What is the publication system for?

The overwhelming variety of current forms reflects the substantial variation in what is expected of peer review. Some of these expectations relate closely to diverging purposes of scientific publishing, which have also shifted over time and are more disparate than one might expect. At first, the main purpose of scientific journals was to settle priority claims, as a social device to establish and maintain intellectual recognition. Specifically using journals for the publication of essentially new knowledge is a relatively recent phenomenon [41, 73]. The main motivation for the prototype of the modern scientific manuscript was ‘the establishment and maintenance of intellectual property. It was the need which scientists felt to lay claim to newly won knowledge as their own, the never-gentle art of establishing priority claims’ [26]. This original purpose of journals became even more apparent in the system of *pli cacheté* that was in place in many journals during the eighteenth, nineteenth and even twentieth century [34]. In this system, authors sent their manuscripts to journals in sealed envelopes, to be opened only at the author’s request. This allowed researchers to submit discoveries about which they were uncertain, while allowing them to claim priority in case other researchers wanted to publish the same or very similar results [34].

Besides settling priority issues and providing due credit to authors, scientific publishing has given rise to three other major expectations. The first is to facilitate the exchange of knowledge and ideas among scholars working in the same narrow field, providing the specialised communication on which research progress depends. The second is to form a constantly evolving historical archive of scholarly thought [106]. The third is to provide a hierarchy of published results based on peer-defined excellence [11, 20, 106, 114]. Or, more briefly stated: ‘In their ideal, journals do not just transmit information; they filter, evaluate, [store] and unify it’ [67].

Peer review plays a major role in two of these functions, namely in facilitating the exchange of ideas among scholars and providing a hierarchy of published results. Firstly, regarding the exchange of knowledge there ‘slowly developed the practice of having the substance of manuscripts legitimated, principally before publication although sometimes after, through evaluation by institutionally assigned and ostensibly competent reviewers’ [119]. As such, peer review is ‘the instrument for ensuring trustworthiness’ in science [20]. Kassirer and Campion explained that the review process ‘is probably best described as an intellectual exercise to detect flaws in experimental design, presentation, interpretation, and the overall importance of a study; at a certain point a manuscript reaches the rejection threshold, which tips the editorial scale toward its rejection’ [60]. That peer review plays a pivotal role in validating research and is widely accepted [12, 15, 90, 113, 114]. This could be the most important aspect of scientific publishing. ‘Ensuring the accuracy and quality of the information contained in a manuscript as well as the clarity of the writing and quality of the presentation is far more important and in some cases crucial’ [106]. The role of quality assurance is attributed to all involved in the review process, not only to reviewers, but specifically also to editors [43].

Secondly, academic publishing provides a hierarchy of published results. Peer review is particularly instrumental in sustaining this hierarchy, by establishing a continuum ranging from top-tier journals to outlets of lower status. An interesting example, in which this expectation of peer review becomes particularly visible, is the mathematics ‘*arXiv* overlay’ journal *SIGMA* (*Symmetry, Integrability and Geometry: Methods and Applications*). This electronic journal, does not ‘publish’ or archive its own articles, but merely adds a signature to articles on *arXiv*, after having reviewed them [102]. As such, the journal does not facilitate the spread or storage of knowledge, but rather assesses articles’ quality and classifies them as sound science. Such classification distinguishes reviewed articles from other manuscripts on arXiv, thereby raising them in the hierarchy of published results. This is not merely an epistemological exercise, but also a quest for recognition of published manuscripts. ‘Peer reviewed publications’ increasingly serve as the basis of research evaluation, be it in grant applications, organisational audits, job interviews or tenure decisions (e.g. [53]). Therefore, elevating manuscripts from the status of preprints to peer reviewed articles serves as a mechanism that not only warrants quality, but also establishes a form of recognition and credit.

Given this hierarchical allocation of recognition, the content of review criteria has become increasingly contentious. Questions arise regarding whether journals merely judge adequacy, consistency and methodological accuracy (e.g. the *PLoS* format), or whether they also account for relevance, perceived impact or usefulness to future research. As a result, tensions have arisen regarding the expectations of what peer review can establish.

Thirdly, the academic publishing system is expected to provide equal and fair opportunities to all participants. As was indicated in the section ‘Main text—the historic development of peer review’, due to the central role peer review has played in its development, this major expectation evolved more gradually [46, 95]. Equal assessment opportunities required submitted manuscripts to be judged on content only, without attention to circumstantial information such as the authors’ affiliation, gender or background. Here, referring to peer-reviewed articles in research career assessment is crucial.

A fourth major expectation of the academic publishing system, and of peer review in particular, emerged in a debate regarding the system’s effectiveness in tracing misconduct. Despite the recognition of peer review’s crucial role in ensuring the accuracy and quality of scientific work, since the late 1980s its capacity to detect fraud has been a growing concern [93]. The discussion was fuelled by reports on major scandals in science, followed by substantial public outcry, including on the Darsee and Baltimore cases [67, 68, 107]. Under the threat of intensified congressional involvement in the USA, the scientific community used the peer review system as one of their main defence arguments. Former National Academy of Sciences (NAS) president Philip Handler called the problem ‘grossly exaggerated’ and expressed complete confidence in the existing system ‘that operates in an effective, democratic and self-correcting mode’ [51]. Similarly, National Institutes of Health (NIH) director Donald S. Fredrickson testified ‘misconduct was not and would never be a problem because of scientific self-regulation’ [51]. In this context, the late 1980s started to exhibit the first major signs of peer review being put forward as a means of safeguarding the scientific enterprise from fraud and misconduct.

However, this argument received criticism from the outset [44, 63, 67]. In the founding days of scientific societies and scientific journals in the seventeenth century, general consensus maintained that the responsibility to guarantee the credibility and soundness of the research record did not lie with the professional society or the publisher [66, 74]. Editors and publishers who still agree that ‘the peer review system was never designed to detect fraud’ [67], implicitly rely on other institutions and whistle-blowers to detect fraudulent data or plagiarised material [116].

Regarding journals’ responsibility to act against misconduct, several actors arrived at different opinions. Even though many journals introduced some measures to address misconduct, for example by issuing retractions and corrections, many believed that more should be done, especially in journals taking a gatekeeper role. In the same period, mainly driven by considerable increases in subscription and submission fees, librarians and authors became more demanding regarding the validity and integrity of published research. At the 1989 annual meeting of the Society for Scholarly Publishing, Hendrik Edelman of Rutgers University declared to generous support of fellow librarians that “given the high costs of subscriptions, publishers should guarantee ‘fraud-free’ products” [67]. The dramatic price increases resulted in heightened agitation for quality control, which was later reinforced by other scholars and librarians [97].

### Tensions regarding peer review and research integrity

The expectation that publishers should be responsible for ensuring the integrity of the scientific literature comes from two sides. Firstly, politicians and funding agencies demand their money be put to good use and thus insist on quality control for the work they finance. From this perspective, peer review plays a role in public accountability. Secondly, authors and librarians increasingly demand value for money, given the high submission and subscription fees of academic journals. Peer review then becomes a matter of product quality.

Despite this twofold call for editors and publishers to take responsibility, many actors, primarily editors and publishers themselves, express disquiet about peer review’s ability to detect fraudulent research. This became strikingly clear in [114] seminal work on the peer review system in which she argues that ‘the underlying strength of editorial peer review is the concerted effort by large numbers of researchers and scholars who work to assure that valid and valuable works are published, and conversely, to assure that invalid or non-valuable works are not published’. At the same time, just a few paragraphs later, she asserts: ‘Fraudulent behavio[u]r on the part of a researcher has not been discussed, primarily because of the limited ability of reviewers or editors to identify fraudulent activities or fabricated data’ [114]. This clearly points to the tension between actors’ desires and expectations regarding the peer review system and the abilities that can reasonably be attributed to it.

In spite of such diverging expectations, some of the current innovations clearly move towards peer review as a factor in improved research integrity. The novel pilot by *Research Square,* providing badges for ‘research with integrity’, arguably indicates that peer review *can* detect fraudulent behaviour if it is specifically designed to do so [81, 100]. In addition, different forms of fraudulent behaviour should be properly differentiated. As has been noted before, it is notoriously difficult for peer reviewers to detect cases of intentional data manipulation or fabrication. However, one can expect several kinds of questionable research practices that are thought to be much more common [59, 75] to be detected by reviewers, as in cases of spin, inappropriate use of statistical analysis or data cooking. In addition, the use of software tools to detect (self-)plagiarism [55], image manipulation and poor statistical analyses has recently increased the detectability of outright misconduct. Detecting these forms of misbehaviour might not reasonably be expected of a single peer reviewer, but can increasingly be expected from *the peer review process*.

## Conclusions

Our review demonstrates the remarkable diversity in contemporary models of peer review. Ever since its establishment, peer review has developed into a wide and expanding variety of forms. The development of review forms can be systematised along four dimensions: (i) the selection conditions, including the timing of the review and its selectiveness; (ii) the identity of and interaction between the actors involved; (iii) the levels of specialisation within the review process; and (iv) the extent to which technological assistance has been implemented in the review system. These four dimensions cover an array of peer review processes than can map both the historic and current forms of peer review, and suggest some axes of possible future development. In addition, this classification can serve as the basis for future empirical research assessing the quality, effectiveness or feasibility of the diverse peer review forms.

Many of the recent innovations have come about as a response to shifting expectations of what peer review can or should achieve. Whereas the post-war dissemination of the system was presented as a form of quality-guarantee, it later responded to concerns regarding inequality in science, the efficiency of the publication system and a perceived increase in scientific misconduct. Currently, four major expectations of the peer review system can be distinguished: (i) assuring quality and accuracy of research, (ii) establishing a hierarchy of published work, (iii) providing fair and equal opportunities to all actors and (iv) assuring a fraud-free research record. Different peer review formats will be preferred, depending on which of these expectations take precedence, as not all of these expectations can be easily combined. For example, a hierarchy of published work through a review process that favours highly relevant, high-impact research can jeopardise equal opportunity, and potentially even accuracy or integrity, as authors go to extreme lengths competing for attention at the top.

To date, very little systematic research has investigated whether peer review can live up to these differing expectations. There is limited evidence on peer review’s capacity to guarantee accurate and high-quality research. Additionally, the potential of peer review to distinguish between possibly relevant and seemingly irrelevant research, or between fraudulent and non-fraudulent research, has not been adequately studied. This leaves a clear knowledge gap to be addressed in future empirical research. Our classification of review forms can constitute a useful tool to set up such comparisons between review practices.

The existing discrepancy between what some expect of the system and what others believe it is capable of has led to several current tensions. Most notably, the expectation that the peer review system should be used in gatekeeping to prevent erroneous or fraudulent research is problematic. Many have blamed peer review for not properly detecting erroneous research; however, simultaneously, others claim it was never designed to do so. Recent new developments and tools in peer review suggest that it is increasingly possible to detect and filter erroneous or fraudulent research in the peer review process. However, more research is needed to investigate the extent to which these innovations can live up to the expectations.

Meanwhile, some of the fraud detection innovations in peer review seem to shift the modalities of knowledge validation. Whereas peer review used to rely on the inter-subjectivity of colleagues to check the objectivity of research, currently, statistics scanners or image-checkers permit more automated judgement in peer review, which aims to reduce human judgement. From inter-subjective checking, the focus is shifting towards more mechanical forms of objectivity, with automated discovery as an uncomfortable asymptote [22].

These tensions about peer review’s expectations and abilities point to more fundamental shifts in ambitions for the scientific publication system. At first, the scientific literature was primarily perceived as a large (public) library containing reports on scientific research, review papers, discussion papers and the like. While this view still prevails, we would argue that an additional frame has appeared, which presents the scientific literature as a database of accurate knowledge or ‘facts’. This new frame, which seems specifically attractive to those holding realist and positivist views of knowledge, is witnessed, for example, in the belief that ‘inaccurate knowledge’ should be retracted from the literature. In the library frame, questioned research was addressed through further publications, referencing and commenting on earlier publications, without removing them. Propositions and knowledge claims, as well as their denials, co-existed in an inter-textual universe of scientific knowledge claims—some more, some less veracious. The publication system as a database insists on removing erroneous records and replacing them with newer, corrected versions through innovative technologies such as corrections, retractions, statistics-checks, or post-publication reviews, facilitated by the digital revolution in publishing. The publication system as database creates new expectations about a body of reliable knowledge, including the possibility of meta-studies or systematic reviews, which are in turn used as arguments to shift further towards a database model. Seemingly technical innovations in the peer review system could therefore be signs of far more fundamental shifts in notions of objectivity or the status of the knowledge contained in ‘the scientific literature’.

## Abbreviations

BMC: BioMed Central
BMJ: British Medical Journal
EMBO: European Molecular Biology Organization
NAS: National Academy of Sciences
NIH: National Institutes of Health
PLoS: Public Library of Science
SIGMA: Symmetry, Integrability and Geometry: Methods and Applications
