# Correction to: The changing forms and expectations of peer review

**DOI:** 10.1186/s41073-018-0058-y

**Published:** 2018-11-14

**Authors:** S. P. J. M. (Serge) Horbach, W. (Willem) Halffman

**Affiliations:** Faculty of Science, Institute for Science in Society, Radboud University Nijmegen, P.O. Box 9010, Nijmegen, 6500 GL The Netherlands

## Correction to: Research Integrity and Peer Review (2018) 3:8 10.1186/s41073-018-0051-5

Following publication of this article [1] it was brought to our attention that we omitted to provide credit for Table 1. While the content of the table and the systematization of blinding in review have been referenced in the text as coming from [2], the credit line for Table 1 should have been added as follows: “Reproduced with permission from [2] licensed under a CC BY-NC-ND 3.0 License”. The original publication of this article has been corrected accordingly.
